# Development and Validation of a Dynamically Updated Prediction Model for Attrition From Marine Recruit Training

**DOI:** 10.1519/JSC.0000000000003910

**Published:** 2021-01-15

**Authors:** Iris Dijksma, Michel H.P. Hof, Cees Lucas, Martijn M. Stuiver

**Affiliations:** 1 Amsterdam UMC Location AMC, Epidemiology and Data Science, Master Evidence Based Practice in Health Care, Arizona, Amsterdam, the Netherlands; and; 2 Defense Health Care Organization, Netherlands Armed Forces, Utrecht, the Netherlands

**Keywords:** dropout, monitoring, landmarking

## Abstract

Dijksma, I, Hof, MHP, Lucas, C, and Stuiver, MM. Development and validation of a dynamically updated prediction model for attrition from Marine recruit training. *J Strength Cond Res* 36(9): 2523–2529, 2022—Whether fresh Marine recruits thrive and complete military training programs, or fail to complete, is dependent on numerous interwoven variables. This study aimed to derive a prediction model for dynamically updated estimation of conditional dropout probabilities for Marine recruit training. We undertook a landmarking analysis in a Cox proportional hazard model using longitudinal data from 744 recruits from existing databases of the Marine Training Center in the Netherlands. The model provides personalized estimates of dropout from Marine recruit training given a recruit's baseline characteristics and time-varying mental and physical health status, using 21 predictors. We defined nonoverlapping landmarks at each week and developed a supermodel by stacking the landmark data sets. The final supermodel contained all but one a priori selected baseline variables and time-varying health status to predict the hazard of attrition from Marine recruit training for each landmark as comprehensive as possible. The discriminative ability (c-index) of the prediction model was 0.78, 0.75, and 0.73 in week one, week 4 and week 12, respectively. We used 10-fold cross-validation to train and evaluate the model. We conclude that this prediction model may help to identify recruits at an increased risk of attrition from training throughout the Marine recruit training and warrants further validation and updates for other military settings.

## Introduction

Whether fresh marine recruits thrive and complete military training programs, or fail to complete, is dependent on numerous interwoven variables. More than 50% fail to complete Marine recruit training, and nearly 1 of the 4 dropouts do so due to musculoskeletal injuries (7). Factors consistently affecting the risk of dropout include body composition, baseline fitness, injury history, smoking, and psychological resilience (8,11,19). At the Marines Training Center (MOC) of the Royal Netherlands Marine Corps (RNLMC), a digital recruit monitoring survey (RMS) is used weekly to measure the status of well-being and recognize declines in an individual or group.

The RMS is one of many investments of the MOC to innovate enlistment, selection and preparation of recruits, as well as to improve monitoring, training, and recuperation. It is applied by embedded monitors with a physical therapy background to carefully monitor recruits during Marine recruit training. The monitors collaborate closely with the medical department (i.e., general practitioner and physical therapist), sports instructors, and the military training staff. These professionals are all part of the same team dedicated to training the recruits to the highest achievable level. The RMS contains self-report items regarding physical, psychological, and social psychological concepts. This includes musculoskeletal pain, coping strategies, motivation, performance ratings, sleep quality ratings, self-esteem, physical and mental fitness, and general health estimations. Mental challenges, fatigue, and musculoskeletal discomfort are more of a rule rather than an exception during the Marine recruit training but—obviously—these challenges and symptoms vary over time.

To date, the RMS has been used to inform training staff based on a pragmatic approach without applying formal algorithms to the acquired data. This approach may discard potentially valuable information because it does not take into account the interaction of the covariates with baseline characteristics and the dynamical changes over time. The interpretation and hence effectiveness of the RMS might be improved by using the information in more formal algorithms to detect recruits at a high risk of dropout throughout their training. Training staff could then act accordingly.

Two previously developed prediction models for the prediction of dropout from military training have been published. One of these models was derived in the RNLMC (3,11). These models include physical characteristics such as body fat percentage and body mass index, as well as self-reported measures such as self-estimated health and self-confidence. Although both models showed promising accuracy for the identification of recruits at a high risk for dropout, they have not been externally validated (4). More importantly, both models used baseline measurements alone. As factors such as motivation, sleep quality, and perceived physical fitness are prone to variation over time, the risk of dropout due to these factors varies as well. We hypothesized that a time-updated model based on landmarking (17), using baseline characteristics as well as repeated self-reported health status, might increase the accuracy and practical value of the model. With landmarking, the follow-up period is divided into several intervals. For each landmark, the probabilities can then be estimated by fitting a survival model to the data from the individuals still in training at that moment, thus accounting for changes in predictor status over time (15).

The objective of this study was to develop and validate a prediction model allowing for dynamically updated estimates of conditional dropout probabilities for attrition from a 24-week Marine recruit training using mental and physical health predictors.

## Methods

### Experimental Approach to the Problem

With a view to the implementation of the prediction model, all procedures in data collection and administration were conform to the usual procedure, i.e., recruits are asked once weekly by email to respond to the RMS digitally. All predictors were defined a priori through selecting biologically relevant variables, which have been suggested to be associated with the risk of musculoskeletal injuries and attrition from military training (3,6,11,12,19).

### Subjects

We undertook a landmarking analysis using prospectively registered data of all recruits starting 24-week marine recruit training (MRT) from January 2016 to August 2018. The minimum age requirement for employment is 17 years and 6 months, with a maximum age of 27 years and 11 months. The minimal height is 1.65 m and minimal body mass is 65 kg. Data were extracted from existing databases of the MOC, Rotterdam, the Netherlands. We collected baseline demographics, baseline fitness, weekly measured mental and physical health status, self-reported musculoskeletal pain, and attrition status of all recruits from 8 consecutive Marine recruit training cohorts. All subjects in these cohorts were men. Starting from on average 90 recruits per cohort with a presumed dropout rate of 50% and 22 a priori determined predictors, data of 5 cohorts were needed to achieve a predictor:event ratio of 1:10. However, because at the time of model development data of 8 cohorts were available, we chose to use all available data to achieve a 1:17 predictor:event ratio (14).

The Medical Research Ethical Committee of the University Medical Center Utrecht, the Netherlands, confirmed that the Medical Research Involving Human Subjects Act does not apply to this study (protocol number: 17-502/C) and waived the study from formal approval. Ethical standards adhered to the World Medical Association's Declaration of Helsinki (10). All subjects signed writen informed consent, authorizing to use their anonymized data for scientific purposes.

### Procedures

Baseline characteristics included body composition (height, body mass, and body fat percentage) and level of education. Baseline physical fitness was measured through the defense physical fitness test, which included a Cooper test (12-minute run for distance) and maximal repetitions of push-ups and sit-ups each in 2 minutes. This test was taken to conform to usual practice, by experienced military sports instructors. Minimal fitness requirements for entry to Marine recruit training include a minimum of 2.7 km on the Cooper test, 30 push-ups, and 30 sit-ups. For this prediction model, we chose a parsimonious set of 6 mental and physical health concepts of the RMS, represented by 6 statements including *I feel in a good health; I felt motivated last week; I do not experience muscle soreness or muscle stiffness; In the past week I slept well; I feel mentally fit; I feel physically fit.* Recruits are asked once weekly to respond to these statements on a 10-point Likert-scale. Self-reported musculoskeletal pain, in any area, was also measured by marking a location and rating the severity on a Numerical Pain Rating Scale (NPRS) from 0 to 10. We dichotomized this variable (“yes” if any location was marked with a corresponding NPRS score and “no” if no injuries were reported) for further analyses, retaining this state (experienced any injury/did not experience any injury) over subsequent time.

The primary outcome for this study was attrition from Marine recruit training that was registered by the secretary initial military training courses of the MOC. In case of dropout, the date and reason (i.e., discharge on individual request, injury, military competences, or other reasons) were also registered. In addition, an embedded monitor sent the recruit an exit survey. We extracted 5 mental and physical health concepts of the exit survey to explore the self-reported reasons for dropout in those domains. Recruits were asked to rate these statements on a 10-point Likert-scale. Concerned statements were as follows: *My preparation was insufficient; Apparently my physical fitness is lacking; I found it too hard mentally; I stopped on the advice of the military physician; Injuries forced me to quit*.

### Statistical Analyses

All analyses were performed using R, version 3.5.2 (13). Reasons for dropout were described as frequencies. In case of dropout in the first 4 weeks, responses on the exit survey at or above 7 were described as the main reason(s) for early dropout.

For the time-updated prediction model, we used a Cox proportional hazard landmark approach for which we defined nonoverlapping landmarks at each week. The training took 24 weeks, thus, we were able to identify S = 23 landmarks. For each landmark time, we created a data set including all recruits who were still in military training up to that landmark time point. We defined a supermodel by stacking the landmark data sets (18). The hazard for individual *i* at landmark s = 1, …S was defined as follows:

$$h\left(t\text{|}Z\left(s\right),s\right)=h_{0}\left(t\right)\text{exp}\left(Z_{i}\left(s\right)\left({\text{β}}_{1}+{\text{β}}_{2}s\right)+{\text{X}}_{i}\text{γ}\right),$$

where $h_{0}\left(t\right)$ was the baseline hazard function, $Z_{i}\left(s\right)$ was the updated values of the time-varying mental and physical health status of individual *i* at the start of landmark *s*, and X_i_ was the baseline characteristics of individual i at the start of the training with corresponding regression coefficients γ. Note that the hazard ratios (HRs) of the time-varying characteristics were allowed to change over landmarks. Parameter estimates of the models as obtained in the imputed data sets were pooled using Rubin's rules and reported as HRs with a 95% confidence interval (95% CI). A detailed description of the landmarking approach for dynamic prediction of survival is provided by van Houwelingen and Putter (18).

#### Missing Data

In case of missing baseline measurements, we used single mean imputation to impute continuous normally distributed baseline characteristics to optimize the sample size and prevent omitting useful information (20). Missing data in the weekly measured time-varying mental and physical health status were imputed using the “last observation carried forward” (LOCF) approach (17), after checking for appropriateness. If it was not possible to impute the mental and physical health status using LOCF for a particular landmark, this landmark was removed from the analysis. For instance, this meant that with missing observations at the first landmark, the follow-up period of this individual started at the landmark at which the time-varying covariates were first observed (i.e., left truncation).

#### Model Validation

We used 10-fold cross-validation to train and test the prediction model and repeated this procedure 500 times. In 10-fold cross-validation, subjects of the original sample are randomly partitioned into 10 equal size subsamples. Of the 10 subsamples, a single subsample is retained as the validation data for testing the model, and the remaining 10−1 subsamples are used as training data.

#### Model Assessment

We assessed the discriminative ability of the model (c-index) based on the area under the time-dependent receiver operating characteristic (ROC) curve (AUC) (2,9). In addition, AUC values were calculated for each landmark s for the prediction of attrition from training at week 1, week 4 (because most of the dropout occurs during the first month in training), and week 12 (longer term follow-up half way in training). We also determined time-dependent sensitivity and specificity at the corresponding time points. We defined a reference subject for interpretability of the regression coefficients by averaging the values of the predictor variables. Our reference subject was 23-year-old and 1.80 meters tall, body mass 80 kgs, had 14% body fat, ran 2.8 km on the Cooper test, did 55 push-ups and 55 sit-ups in 2 minutes each, and scored a neutral (5) score on the mental and physical health status. Finally, to quantify the added value of the time-updated approach, we compared the prediction accuracy of the model with a conventional Cox proportional hazard model including baseline variables only (15).

## Results

Data were available from 744 recruits, of whom 408 (54.8%) dropped out from training. Ten (1.4%) individuals dropped out in the first week without any mental and physical health status observations. Therefore, they were excluded from further analysis. From 64 (8%) recruits one or more baseline measurements were missing and imputed by means. In 32 (4%) recruits, the time-varying characteristics were left truncated. Baseline characteristics are described in Table 1.

**Table 1:** Baseline characteristics of the recruits included for model development and model validation.

Variable	All observations, *N* = 744
	Mean ± *SD*
Age (y)	21 ± 2.4
Height (m)	1.81 ± 6.4
Body mass (kg)	77.9 ± 7.9
Body fat %	13.6 ± 3.1
Cooper test (km)	2.87 ± 1.58
Push-ups in 2 minutes	54.2 ± 11.5
Sit-ups in 2 minutes	55.0 ± 7.5
Secondary vocational education (level 2 higher than 1)	Level 1: 184 (25%)
	Level 2: 130 (17%)
	Unknown: 430 (58%)

Of the 408 dropouts, 137 recruits dropped out because of musculoskeletal pain, 240 recruits were discharged on individual request (because of lack of motivation to follow through), and 27 recruits due to other reasons. Self-reported reasons for early dropout (first 4 weeks of Marine recruit training; 22%, multiple options possible, missing data *n* = 25) were as follows: “*I found it too hard mentally” n* = 21, “*Apparently my physical fitness is lacking” n* = 17, “*Injuries forced me to quit” n* = 11, “*I stopped on the advice of the military physician” n* = 8, and “*My preparation was insufficient” n* = 7.

The final time-updated supermodel contained all but one of the a priori selected baseline variables and time-varying covariates to predict the hazard of attrition from Marine recruit training for each landmark as comprehensive as possible. The statement “*I feel mentally fit”* was excluded from the model because most recruits had no measurements (61%) and its variance was close to zero. Hazard ratios with 95% CI of the models with and the without time-updated predictors are presented in Table 2.

**Table 2:** Parameter estimates showing the effect of multivariable factors on the risk of attrition from 24-week Marine recruit training.*†

	Cox proportional hazard landmark supermodel hazard ratio (95% CI)	*p*
1		
Height (m)	0.674 (0.064–7.143)	0.743
2		
Body mass (kgs)	0.957 (0.938–0.977)	<0.001
3		
Age (y)	0.967 (0.922–1.015)	0.175
4		
Education 1	0.913 (0.71–1.174)	0.478
5		
Education 2‡	0.675 (0.483–0.944)	0.022
6		
Body fat (%)	1.034 (0.991–1.079)	0.125
7		
Cooper test (per km)	0.157 (0.076–0.325)	<0.001
8		
Push-ups (*n*, per 10)	0.984 (0.876–1.106)	0.788
9		
Sit-ups (*n*, per 10)	0.999 (0.852–1.17)	0.987
10		
Health	0.984 (0.799–1.211)	0.877
11		
Motivated	0.655 (0.578–0.741)	<0.001
12		
Muscle soreness	1.053 (0.945–1.173)	0.353
13		
Sleep	1.048 (0.922–1.191)	0.473
14		
Physical fitness	0.792 (0.651–0.964)	0.020
15		
Self-reported MSI	0.673 (0.182–2.489)	0.553
16		
Health: landmark	0.992 (0.971–1.012)	0.418
17		
Motivated: landmark	1.018 (1.005–1.031)	0.007
18		
Muscle soreness: landmark	0.998 (0.987–1.009)	0.713
19		
Sleep: landmark	1.004 (0.992–1.017)	0.507
20		
Physical fitness: landmark	1.007 (0.987–1.027)	0.505
21		
Self-reported MSI: landmark	0.999 (0.909–1.097)	0.984

* CI = confidence interval n = number.

† The first 15 predictors are baseline variables, and variables 16 to 21 represent the regression coefficients of the landmarks.

‡ Secondary vocational education, Level 2 higher than 1, included as dummy variables; MSI, self-reported musculoskeletal injury; hazard ratios for continuous variables refer to one unit change; landmark variables for the time-updated predictors can be interpreted as Health:1, Health:2, Health:3 and so on.

**Figure 1.**
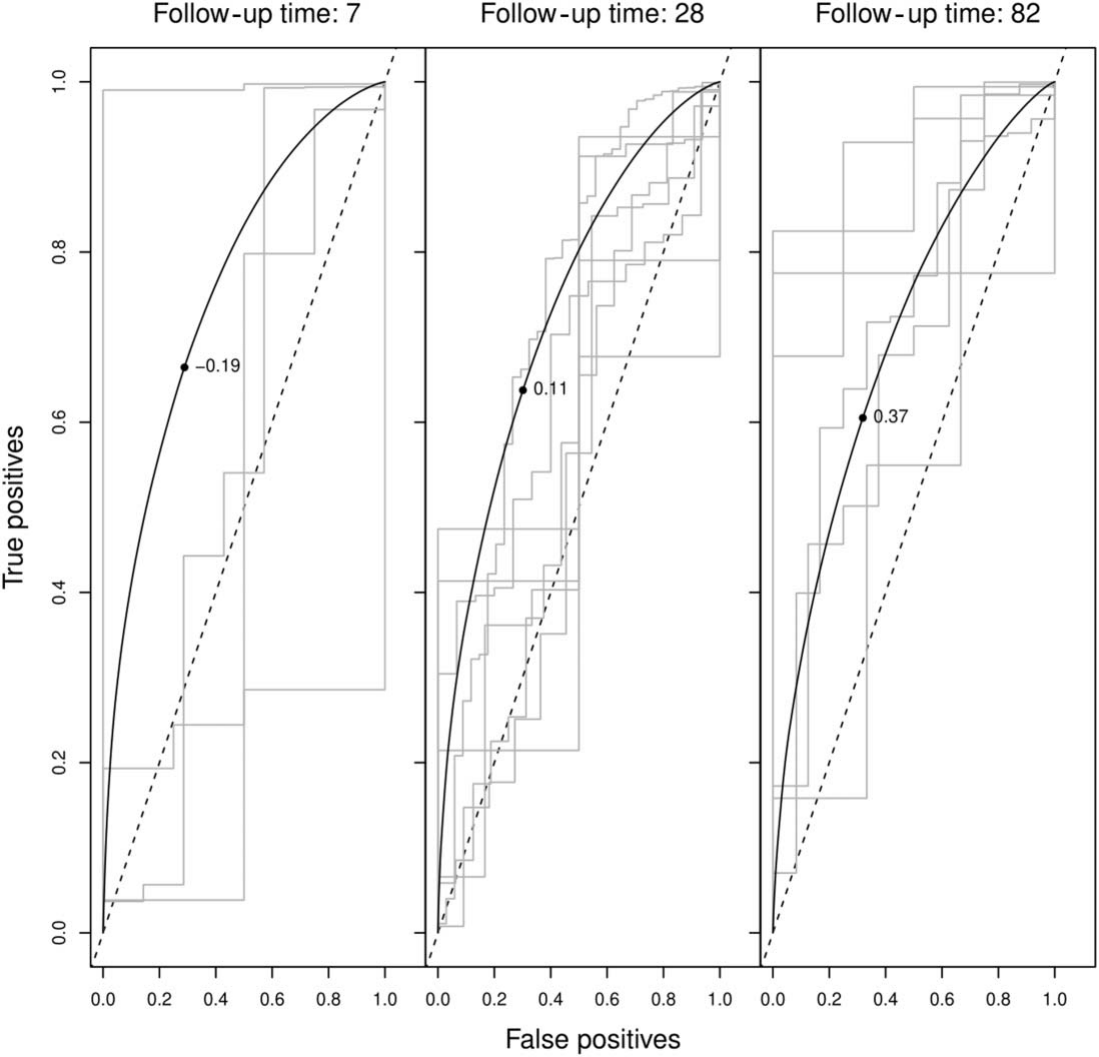
Unsmoothed and smoothed dynamic ROC curves demonstrating the discriminative performance of the prediction model for attrition from Marine recruit training at day 7, 28, and 84 (week 1, 4, and 12, respectively). The accuracy of the model, which is measured by the area under the curve, at week 1, 4, and 12 was 0.78, 0.75, and 0.73, respectively, as depicted by the ROC. The unsmoothed ROCs are from time points that are located in a period of 2 weeks around the time point of the smoothed ROC (i.e., day 28: day 21 to day 35). The raw curves represent follow-up times with one or more events occurring. The numbers in the graph (i.e., −0.19) represent the optimal threshold for the linear predictors (log hazard ratio) at each follow-up time point. *Our reference subject was 23–year-old and 1.80 meters tall, body mass 80 kgs, had 14% body fat, ran 2.8 km on the Cooper test, did 55 push-ups and 55 sit-ups in 2 minutes each, and scored a neutral (5) score on the mental and physical health status. ROC, receiver operating characteristic.

The linear predictor for an individual at each point in time reflects the hazard of attrition from Marine recruit training given a subject's baseline characteristics and updated mental and physical health status. Figure 1 shows the dynamic ROC curves with the thresholds of the linear predictor that leads to the highest sensitivity and specificity at particular follow-up times.

**Figure 2.**
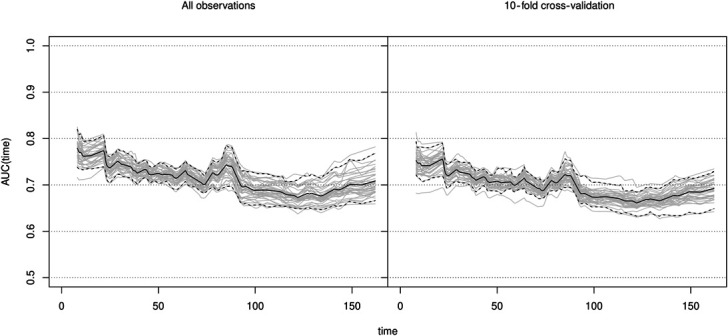
Time-dependent AUC curve, follow-up time in days. Dynamic AUC plots all observations (left) and 500 times 10-fold bootstrap cross-validation (right), representing the accuracy of the model score (linear predictor) under the assumption of proportional hazards.

The AUCs of the landmark supermodel were 0.78 (95% CI 0.74–0.82), 0.75 (0.72–0.77), and 0.73 (0.69–0.75) in week 1, week 4, and week 12, respectively. The time-dependent AUC declined slightly over time as the number of events—dropouts from training—increased. The AUC can be interpreted as the probability that a recruit who drops out on landmark s had a model score (linear predictor) higher than that of a recruit who retains beyond s. The sensitivity and specificity were 66%, 71%, in week 1; 64%, 70% in week 4; and 60%, 68% in week 12, respectively. Figure 2 shows the time-dependent discriminative ability of the Cox model as the AUC over time in days (*t*), in the observed data and corrected for over optimism using 10-fold cross-validation as the AUC over time in days (*t*). The estimated AUC(*t*) function tends to decline over time, but discriminative ability remains fair (>0.70 AUC(t)). The over optimism corrected AUCs in week 1, 4, and 12 were 0.75 (0.72–0.79), 0.73 (0.71–0.75), and 0.71 (0.69–0.73), respectively. This model is programmed in Shiny (5) from R and is available on request. For the use of this model, baseline variables and weekly values of the RMS can be implemented in the Shiny app to obtain individual linear predictors that can be held against the time-dependent threshold to quantify individualized and time-updated risk of dropout from training.

The AUCs of the conventional Cox proportional hazard model including only baseline variables were 0.65 (0.61–0.69), 0.65 (0.62–0.68), and 0.64 (0.61–0.67) for predicting dropout in week 1, week 4, and week 12, respectively.

## Discussion

The purpose of this study was to develop a prediction model to derive dynamically updated estimates of conditional dropout probabilities of an individual in Marine recruit training. Through landmarking, we efficiently used longitudinal data by exploring information from the same individual at several landmarks and incorporating time-updated covariates. The prediction model includes baseline characteristics and time-varying mental and physical status with an AUC of 0.71 (0.69–0.73) in week 12 of Marine recruit training. Although the estimated AUC (*t*) function in the validation data set showed some decline over time, discriminative ability remained fair. By evaluating the model performance using 10-fold cross-validation we demonstrated that the discriminative ability of the model remained >0.70 AUC (*t*). Thus, the model showed no clear signs of overfitting (i.e., there was a limited decrease in AUC) that suggests the model is robust and applicable in practice.

The model confirmed that body mass and baseline physical fitness are strong predictors for dropout from MRT. Notably, the HR for muscle strength (i.e., the maximum number of push-ups and sit-ups that could be completed) seems to be small. However, this might be a consequence of the small range in observations in this sample and the fact that all recruits met the minimum fitness criteria. In addition, one should keep in mind that the HR reflects a change in risk per 10 repetitions change (10 push-ups/10 sit-ups more). Thus, with further decrease in muscle strength, the individual dropout risk still increases accordingly.

One of the previously published models also included “trainers' judgment”—which was based on the degree to which the trainers believed the recruits showed the necessary effort (“military quality”) and capacity to complete the training—which seemed to be a strong predictor (3). The effect also held for the judgments made after the first 2 weeks, indicating very early predictions. We speculate, however, that the strong effect of trainers' judgment is an example of the Golem effect—which relates to the negative effects of self-fulfilling prophecies, distortions in information processing, biases, and stereotypes (1). The Golem effect describes the phenomena that people of whom little is expected perform less well, resulting in even lower expectations and performing even worse (1). We believe that this effect is undesirable and should be minimized by fostering a growth mindset of recruits as well as their trainers. In such a situation, subjective judgment is likely a less useful predictor. Hence, we did not include subjective trainers' judgment and instead focused on recruits' self-reported status.

The regression coefficients of the time-updated predictors are close to the neutral value, which is due, in part, to the fact that we identified *S* = 23 landmarks, which resulted in small time-updated effects per landmark. Only the effect of both the baseline value and time-updated effect over the landmarks of the variable “*I felt motivated last week”* —typically prone to variation—was statistically significant. The baseline regression coefficient (0.66, log odds ratio of −0.42) shows that high levels of self-reported motivation at entry military training indicates a lowered risk of dropout from training. In addition, the regression coefficient of the landmarks (1.02, log odds ratio 0.02) indicates that the effect of this covariate weakens as time passes—moving from a log odds ratio of −0.42 at *s* = 1 to −0.40 at s = 2 and so on. Yet, the AUCs of the landmark supermodel compared with the AUCs of the Cox proportional hazard model showed that the addition of the time-updated predictors resulted in a meaningful increase in the discriminative ability of the model meaningfully. Moreover, contrary to previously developed prediction models for attrition in the RNLMC (3) we did not select predictor variables based on the *p* value of their regression coefficients because the risk of overfitting the model using such a data-driven approach is relatively high (16). Thus, we believe that this model will likely yield better predictions of dropout risk compared with previous models. However, external validation should still be performed to confirm the predictive accuracy when the model is applied to new individuals.

In our sample, nearly 60% of recruits did not complete Marine recruit training. Notably, 22% of the dropouts occurred in the first 4 weeks of training. The statement “*It was too hard mentally”* was predominantly reported as the main reason for dropout in the exit survey. However, because the majority was missing (i.e., 316 recruits [61%] had no observations) and the available data contained little variation, the regression coefficient of the time-varying covariate “*I have a good mental fitness”* could not accurately be estimated and was therefore not included in the prediction model. We hypothesize that these values were missing not at random (i.e., the value of the observation caused the missingness of the observation itself). This emphasizes the importance of measuring mental fitness accurately. We suggest substituting this item in the RMS because it does not seem to capture signs of dejection or apathy. Possibly one or multiple items measuring personality traits would be useful and should be investigated for their possible contribution to future updates of the prediction model.

We developed this prediction model to the best of our abilities by making a priori choices of inclusion of variables that are readily available in practice, to prevent developing an overly data-driven or hard-to-implement model. However, various sources of uncontrolled variables raise uncertainties in the interpretations of the outcomes. First, we used data that were previously collected as a usual procedure to monitor the training status of the recruits. Therefore, we had no control over how the data were registered prospectively. Second, by using self-reported measures we relied on the honesty of the recruits on the time-varying variables. Third, there were remarkably few self-reported musculoskeletal injuries in the RMS. As a result, the regression coefficient for this predictor shows strong statistical uncertainty as reflected by the wide CI.

**Practical Applications**

We conclude that the prediction model developed in the current study, which includes baseline characteristics and time-varying mental and physical status, could be useful to identify recruits at an increased risk of attrition from training throughout the Marine recruit training. Using the model and corresponding Shiny app, embedded monitors can detect fluctuations in individual attrition risk by obtaining individual linear predictors that can be held against the time-dependent threshold to quantify individualized and time-updated risk of dropout from training. Subsequently, they can educate and advise training staff to make adaptions both on the group and individual level. Possible strategies to provide timely adjustments in the case of an elevated dropout risk can be individualization of training programs; offering flexible training times for individuals to optimize training adaptations; routine monitoring of training load; and clearly defined flexible updated guidelines for training load and managing adherence to those guidelines. We consider it worthwhile to attempt validation and/or updating of the model in other military settings. Suggestions for further research are investigating the association between personality traits and their association to (early) dropout from Marine recruit training to further strengthen the model and implementation research to assess the performance and impact of this prediction model when applied in daily military practice.

## References

[1] Babad EY Inbar J Rosenthal R. Pygmalion, galatea, and the Golem: Investigations of biased and unbiased teachers. J Educ Psychol 74: 459–474, 1982.

[2] Bansal A Heagerty PJ. A comparison of landmark methods and time-dependent ROC methods to evaluate the time-varying performance of prognostic markers for survival outcomes. Diagn Prognost Res 3: 14, 2019. 10.1186/s41512-019-0057-6 PMC6657082 31367681

[3] Binsch O Banko KM Veenstra BJ Valk PJL. Examining the relationship between mental, physical, and organizational factors associated with attrition during maritime forces training. J Strength Cond Res 29(suppl 1): S187–S191, 2015. 26506186 10.1519/JSC.0000000000001117

[4] Bleeker SE Moll HA Steyerberg EW . External validation is necessary in prediction research: A clinical example. J Clin Epidemiol 56: 826–832, 2003. 14505766 10.1016/s0895-4356(03)00207-5

[5] Chang W. Web Application Framework for R Package “shiny.” 1.4.0. Available at https://cran.r-project.org/web/packages/shiny/shiny.pdf. Accessed August 27, 2020.

[6] Connaboy C Eagle SR Johnson CD . Using machine learning to predict lower-extremity injury in US Special Forces. Med Sci Sports Exerc 51: 1073–1079, 2019. 30985586 10.1249/MSS.0000000000001881

[7] Dijksma I Zimmermann WO Hertenberg EJ Lucas C Stuiver MM. One out of four recruits drops out from elite military training due to musculoskeletal injuries in The Netherlands Armed Forces. BMJ Mil Health 168: 136–140, 2022. 10.1136/bmjmilitary-2020-001420 PMC8961760 32139408

[8] Dimitriou L Lockey J Castell L. Is baseline aerobic fitness associated with illness and attrition rate in military training? J R Army Med Corps 163: 39–47, 2017. 26929111 10.1136/jramc-2015-000608

[9] Heagerty PJ Zheng Y. Survival model predictive accuracy and ROC curves. Biometrics 61: 92–105, 2005. 15737082 10.1111/j.0006-341X.2005.030814.x

[10] Kong H West S. WMA Declaration of Helsinki—Ethical Principles for Medical Research Invol, 2017, 1964. pp. 1–9. Available at: http://eds.b.ebscohost.com/eds/pdfviewer/pdfviewer?sid=a4bced6b-7270-457a-9730-e5a47e439a7a%40sessionmgr105&vid=6&hid=126. Accessed August 27, 2020.

[11] Moran DS Evans RK Arbel Y . Prediction model for attrition from a combat unit training program. J Strength Cond Res 25: 2963–2970, 2011. 21988903 10.1519/JSC.0b013e318212dcf7

[12] Pope RP Herbert R Kirwan JD Graham BJ. Predicting attrition in basic military training. Mil Med 164: 710–714, 1999. 10544625

[13] R Core Team. R. A Language and Environment for Statistical Computing. Vienna, Austria: R Foundation for Statistical Computing; 2015. Available at: http://www.r-project.org/. Accessed August 27, 2020.

[14] Riley RD Ensor J Snell KIE . Calculating the sample size required for developing a clinical prediction model. BMJ 368: m441, 2020. 32188600 10.1136/bmj.m441

[15] Rizopoulos D Molenberghs G Lesaffre EMEH. Dynamic predictions with time-dependent covariates in survival analysis using joint modeling and landmarking. Biometrical J Biometrische Z 59: 1261–1276, 2017. 10.1002/bimj.201600238 28792080

[16] Steyerberg EW Vergouwe Y. Towards better clinical prediction models: Seven steps for development and an ABCD for validation. Eur Heart J 35: 1925–1931, 2014. 24898551 10.1093/eurheartj/ehu207 PMC4155437

[17] van Houwelingen H. Dynamic prediction by landmarking in event history analysis. Scand J Stat 34: 70–85, 2007.

[18] van Houwelingen H Putter H. Dynamic predictions using biomarkers (Chapter 8). In: Dynamic Prediction in Clinical Survival Analysis (1st ed.). Boca Raton: CRC Press, 2011. pp. 121–136.

[19] Walker TB Lennemann LM McGregor JN Mauzy C Zupan MF. Physiological and psychological characteristics of successful combat controller trainees. J Spec Oper Med 11: 39–47, 2011. 10.55460/7ZRU-MW0D 21455909

[20] Zhang Z. Missing data imputation: Focusing on single imputation. Ann Translat Med 4: 9, 2016. 10.3978/j.issn.2305-5839.2015.12.38 PMC4716933 26855945

